# Treatment options and outcomes for delayed scapular anatomical neck fractures: a case report and review of the literature

**DOI:** 10.1186/s13256-024-04424-3

**Published:** 2024-03-09

**Authors:** Farzad Amouzadeh Omrani, Mohammad Khak, Reza Tavakoli Darestani, Sina Afzal, Mojtaba Baroutkoub, Mahdi Aghaalikhani, Hasan Barati

**Affiliations:** 1 Department of Orthopedic Surgery, School of Medicine, Shahid Beheshti University of Medical Sciences, Tehran, Iran; 2 Musculoskeletal Translational Innovation Initiative, Beth Israel Deaconess Medical Center, Harvard Medical School, Boston, MA USA

**Keywords:** Scapula, Anatomical neck, Fracture fixation, Delayed diagnosis, Case reports

## Abstract

**Introduction:**

Scapular anatomical neck fractures are among the most infrequent shoulder girdle fractures. Only seven radiologically confirmed cases of scapular anatomical neck fractures have been documented in the literature to date, of which only one case underwent delayed surgery.

**Case presentation:**

A 34-year-old male Persian patient with morbid obesity was diagnosed with a scapula anatomical neck fracture after a motor vehicle collision. The radiographic assessment of the patient indicated an increase in the scapular glenopolar angle (73.9°). Due to concurrent chest and head injuries, surgical intervention was deferred until 6 weeks following the injury. The posterolateral limited Dupont–Evrard approach was used because of the patient’s extremely high body mass index. Two plates were utilized to achieve stable fixation of the glenoid neck fracture. Following a 1 year follow-up period, complete fracture union was successfully attained, resulting in a constant score of 79.

**Conclusions:**

The most accurate radiographic indicators of these fractures are a superior fracture line located laterally to the coracoid process, a small inferior spike, and an elevated glenopolar angle. The only tendon attached to the glenoid is the long head of the triceps, making these fractures unstable; therefore, surgery is required in the majority of instances. The small size of the fractured component makes stabilization more difficult. Overall, anatomical scapular neck fractures are extremely uncommon and distinguished from other scapular fractures by their unique radiological and biomechanical characteristics. This case highlights the challenges encountered when managing scapular fractures in patients with morbid obesity. The delayed surgical intervention and the choice of surgical approach tailored to the patient’s specific anatomical and physiological considerations proved to be effective in achieving a favorable outcome.

## Introduction

The neck of the scapula is the zone that connects the glenoid (scapula head) to the scapula body. The length, alignment, and angulation of the scapula neck are critical for shoulder function and rotator cuff movements [1]. Because of the rarity of these fractures, there have been substantial conflicts and, in some cases, inconsistencies in the precise classification and definition of these fractures [2]. The scapula anatomical neck is identified as the constricted zone that encircles the glenoid cavity laterally, extending from the glenoid fossa to the coracoid process, whereas the surgical neck is located medially to the coracoid process and is more prone to fracture following traumatic events [3].

Scapular fractures account for only 1% of all body fractures, and 7% of those are scapular neck fractures [4, 5]. The majority of these fractures are caused by high-energy trauma, such as motor vehicle collisions or falls from height. Studies have shown that in 80–95% of cases, patients present with concomitant injuries, particularly thoracic injuries, which require immediate attention and management. Prioritizing the stabilization and treatment of associated injuries may necessitate a delay in the surgical intervention for the scapular fracture [6]. Scapular neck, also mentioned as glenoid neck fractures, are categorized into three types: anatomical neck fractures (ANFs), surgical neck fractures, and trans-spinous fractures [5, 7].

Although there have been several reports of this particular type of fracture in the existing literature, reliable radiological evidence to support some of these claims is limited. Through an extensive literature search, our investigation yielded a limited number of radiologically confirmed cases of scapula ANF. To date, only seven such cases have been documented in the available literature [3, 5, 7–10]. Remarkably, among these cases, only one case underwent delayed surgery, highlighting the scarcity of evidence pertaining to the optimal management approach for this fracture pattern [9]. In this study, we aim to introduce a rare case of concurrent scapular anatomical neck fracture and humeral greater tubercle avulsion fracture, which was complicated by a referral delay of 6 weeks and a high body mass index (BMI). We also discuss the clinical presentation, diagnostic assessment, surgical management, and postoperative outcomes of this scapula anatomical neck fracture. In addition, we review previous case reports of scapular anatomical neck fractures to provide comprehensive evidence to guide the diagnosis, classification, and treatment of scapula anatomical neck fractures.

## Case presentation

### History and assessments

**Fig. 1:**
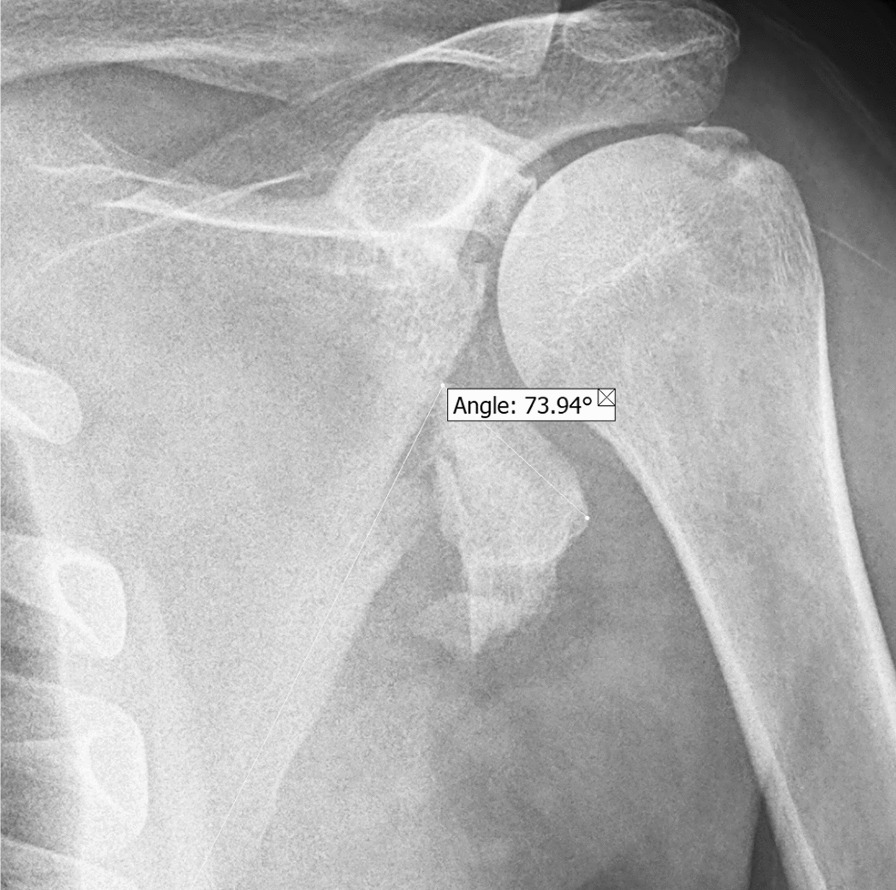
Preoperative X-ray. The humeral greater tubercle avulsion fracture and scapula anatomical neck fracture, along with an increase in the glenopolar angle, are evident in this photo

**Fig. 2:**
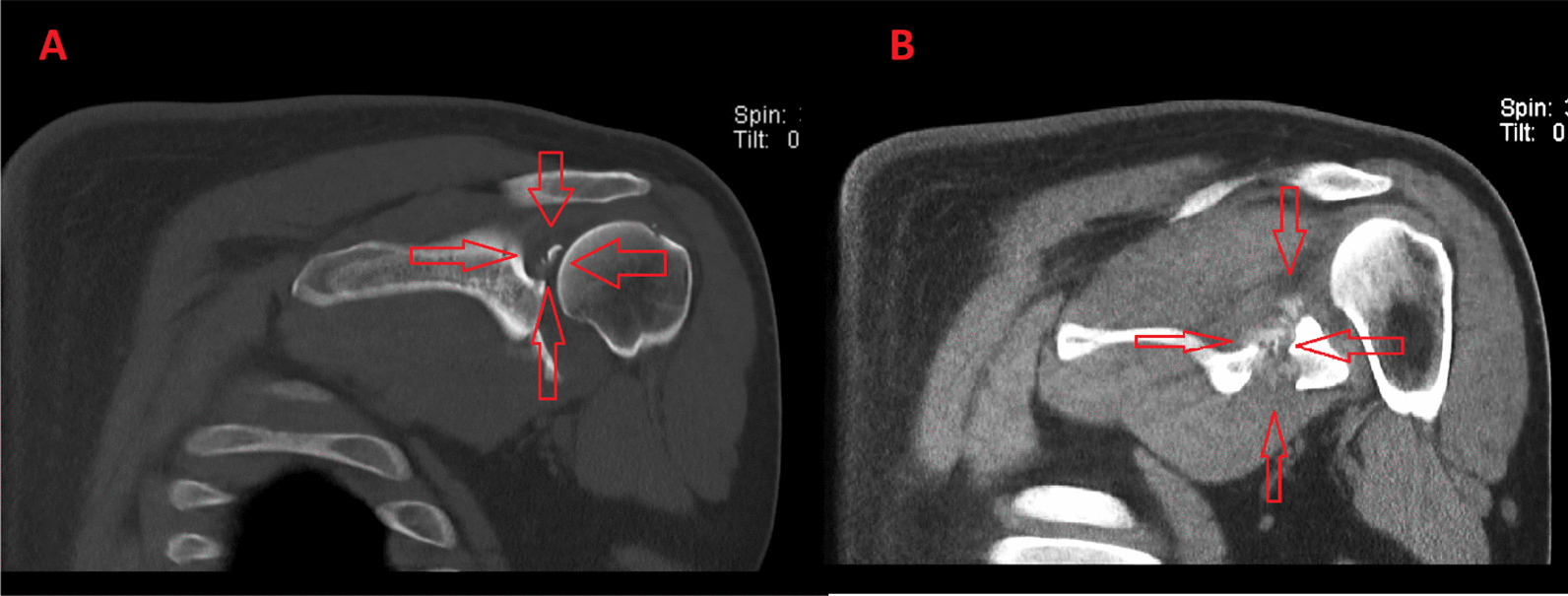
Coronal views of the shoulder CT scan. The red arrows indicate comminution in the region of the superior fracture line (**A**). The red arrows indicate callus formation at the fracture site (**B**)

A 34-year-old morbidly obese male Persian patient was admitted to a secondary care facility following a motor vehicle accident, 6 weeks prior to his referral to our tertiary center. At the secondary center, the general surgeon performed bilateral chest tubes insertion to treat hemothorax resulting from rib fractures sustained in the accident. Additionally, the patient was evaluated by the neurosurgery service for a mild concussion, which required monitoring. After 3 weeks of hospitalization, the patient regained full consciousness and the chest tubes were successfully removed. At this point, he was discharged from the secondary care facility and referred for further evaluation and treatment of the scapula fracture. The patient’s medical history was unremarkable, except for a significantly high BMI of 42 kg/m^2^, indicative of morbid obesity. Upon presentation at our center, a thorough examination of the patient’s neurovascular status revealed no abnormalities. Plain radiographs, including anteroposterior and lateral views, revealed scapula ANF with increased glenopolar angle (GPA) (Fig. 1). Computed tomography (CT) imaging further demonstrated callus formation at the fracture site, along with comminution at the superior part of the fracture line. Additionally, humeral greater tubercle avulsion fracture was also noted (Fig. 2).

### Surgical technique

**Fig. 3:**
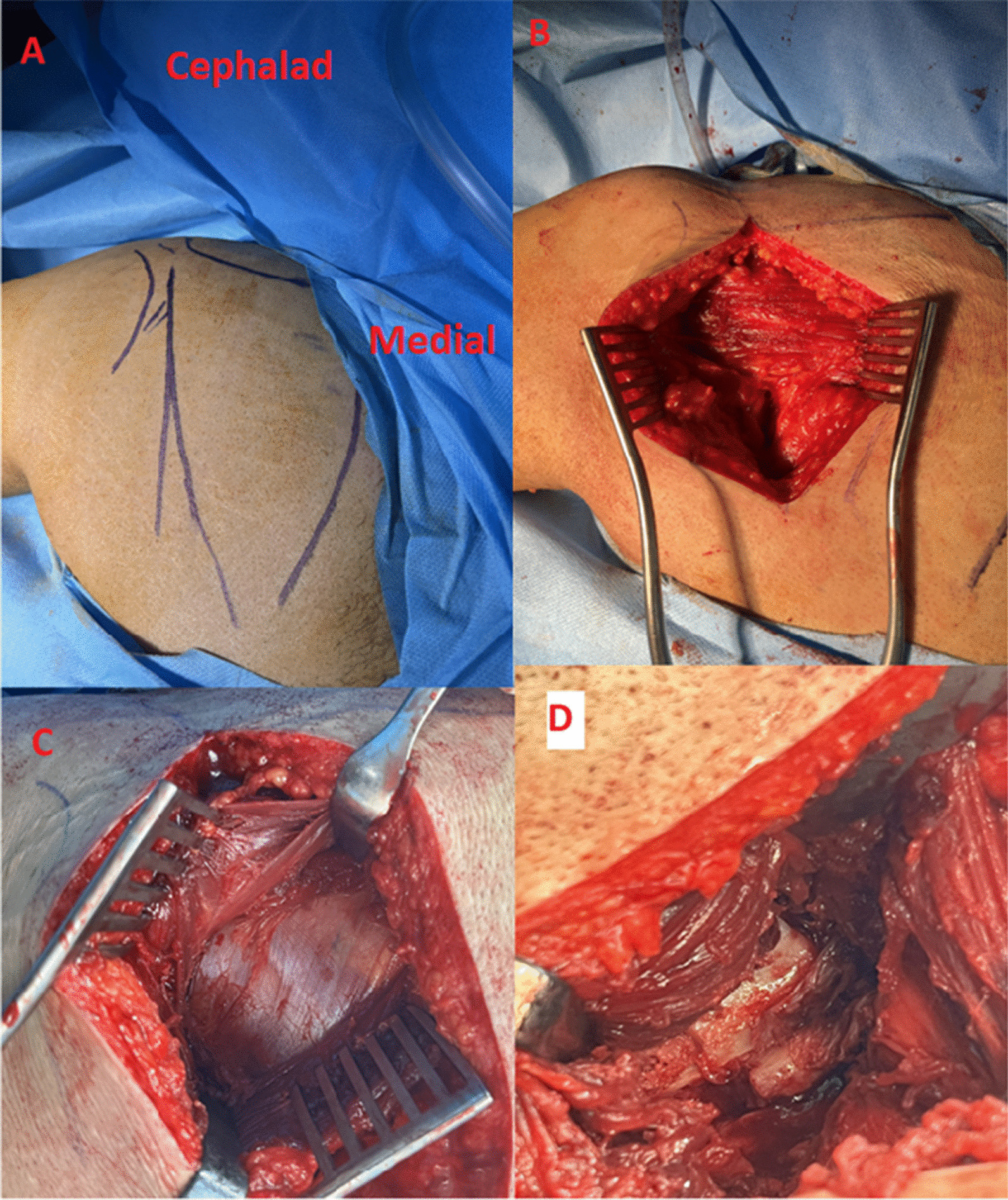
Posterolateral limited Dupont–Evrard approach. The incision commences at the posterior corner of the acromion and extends distally parallel to the lateral border (**A**). The deltoid’s inferior border appears after superficial dissection (**B**). The infraspinatus fascia is located deep within the deltoid muscle (**C**). The scapular neck fracture appears deep in the interval between the infraspinatus and teres minor muscles (**D**)

The patient underwent surgery 6 weeks after the accident. Due to the patient’s morbid obesity, special accommodations were made to optimize surgical access and reduce the potential complications associated with positioning. Large pelvic and thoracic supports were used to allow the patient’s abdomen to hang freely, enabling the diaphragm to move freely and improve oxygenation. The posterolateral limited Dupont–Evrard approach [11] was used because of the patient’s extremely high BMI to minimize the risk of wound-healing complications. This approach used the intermuscular plane between the teres minor and infraspinatus muscles to reach the neck and lateral border of the scapula (Fig. 3).

**Fig. 4:**
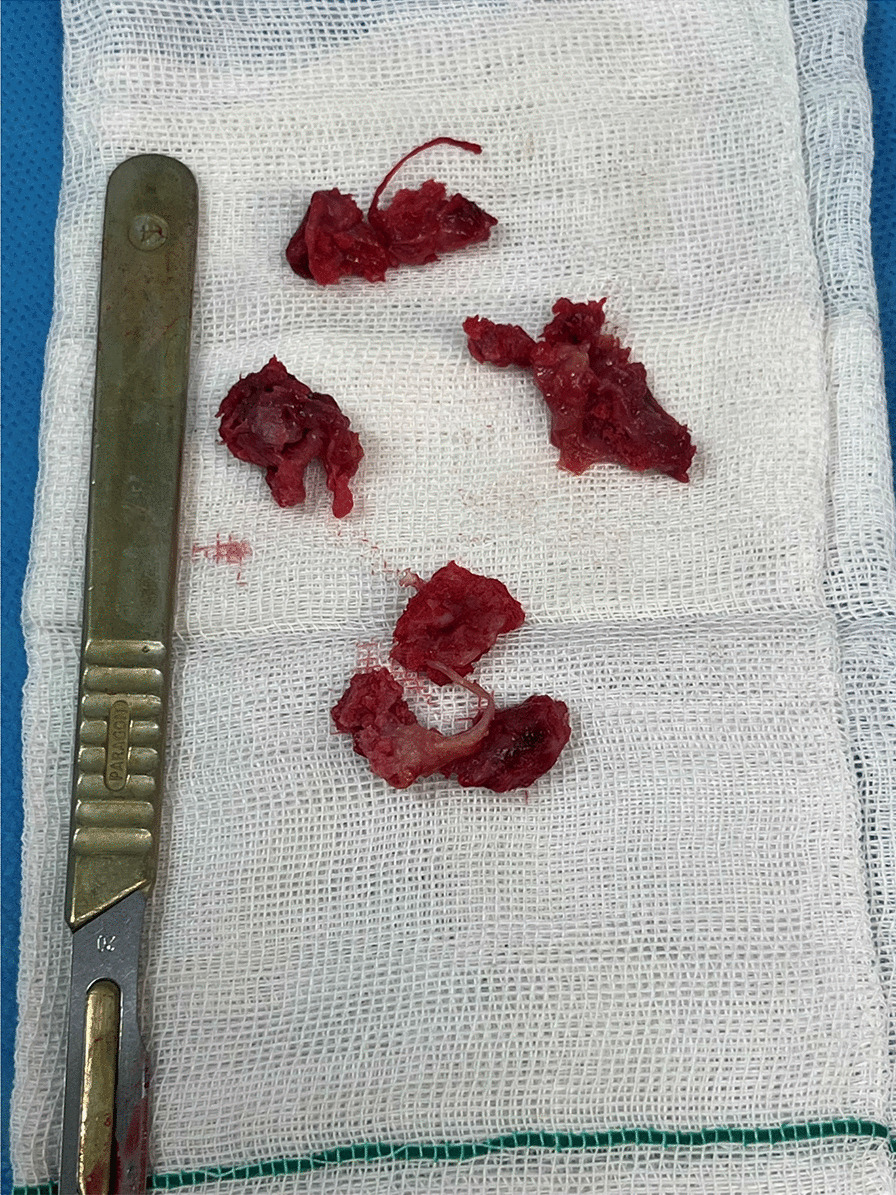
The calluses are excised from the fracture site to facilitate mobilization of the fracture

**Fig. 5:**
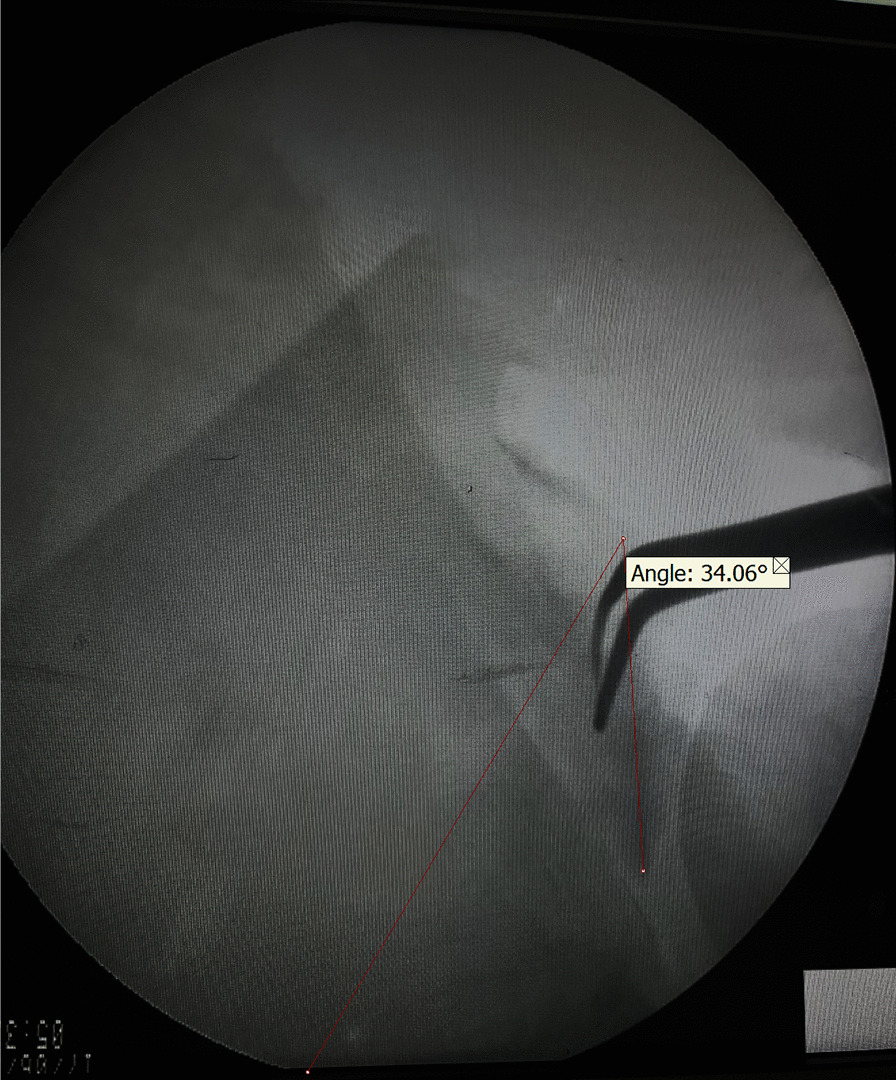
Confirming the glenopolar angle correction and medialization with intraoperative fluoroscopy

**Fig. 6:**
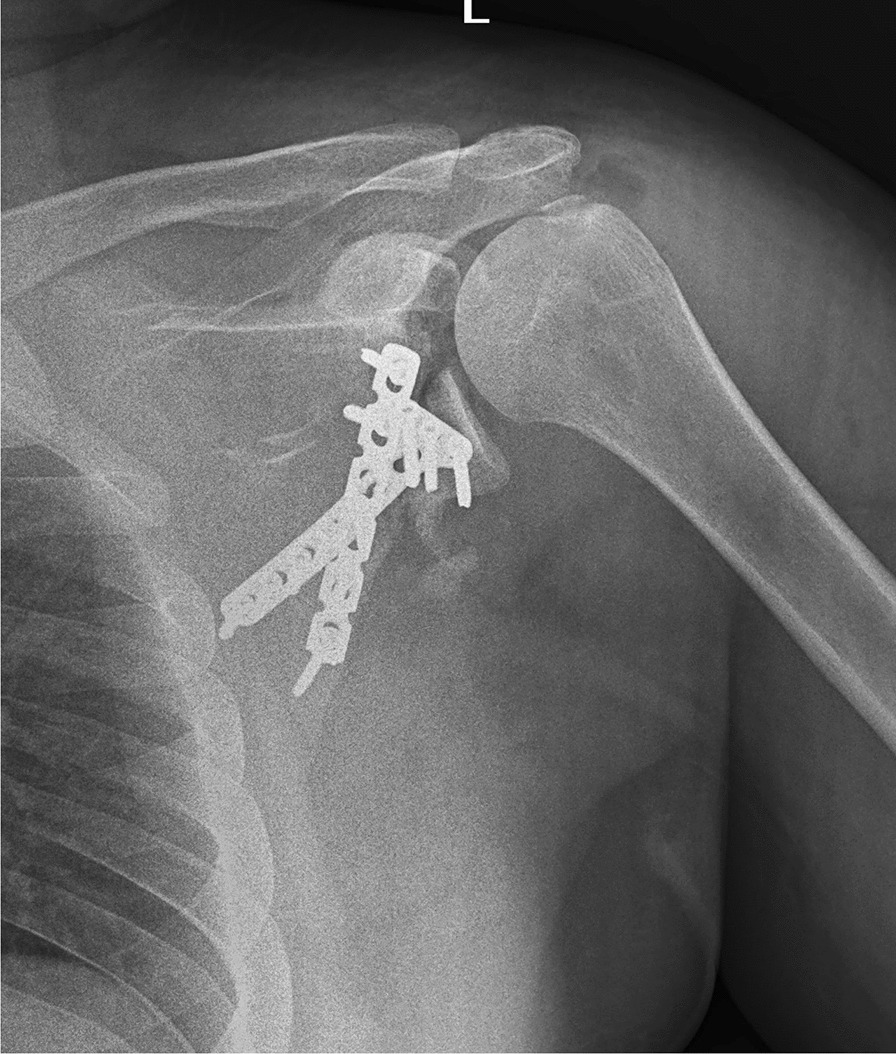
Postoperative shoulder X-ray shows the utilization of two reconstruction and T 3.5 mm plates to fixation of the scapula anatomical neck fracture

With particular attention to preserving the integrity of the circumflex scapular artery and supraspinatus nerve, the callus formed along the fracture line was carefully resected and the glenoid was released and mobilized (Fig. 4). This further allowed for better visualization and manipulation of the fractured segments. Given the deformation and blunting of fracture edges due to chronicity, reduction was conducted under C-arm guidance to achieve an acceptable realignment of fractured fragments, based on the normal radiologic values such as GPA (Fig. 5) [12]. Following confirmation of the reduction, a 3.5 mm low-profile T-plate was employed to secure the anatomical neck in place. Despite the small size of the fractured segment, proper placement of the horizontal portion of the plate in relation to the glenoid fossa enabled the insertion of three screws into the broken fragment. Subsequently, the lateral pillar fracture was fixed using a reconstruction plate. It is worth noting that the avulsion fracture of greater tubercle remained intact due to its minimal displacement (Fig. 6).

### Postoperative care

**Fig. 7:**
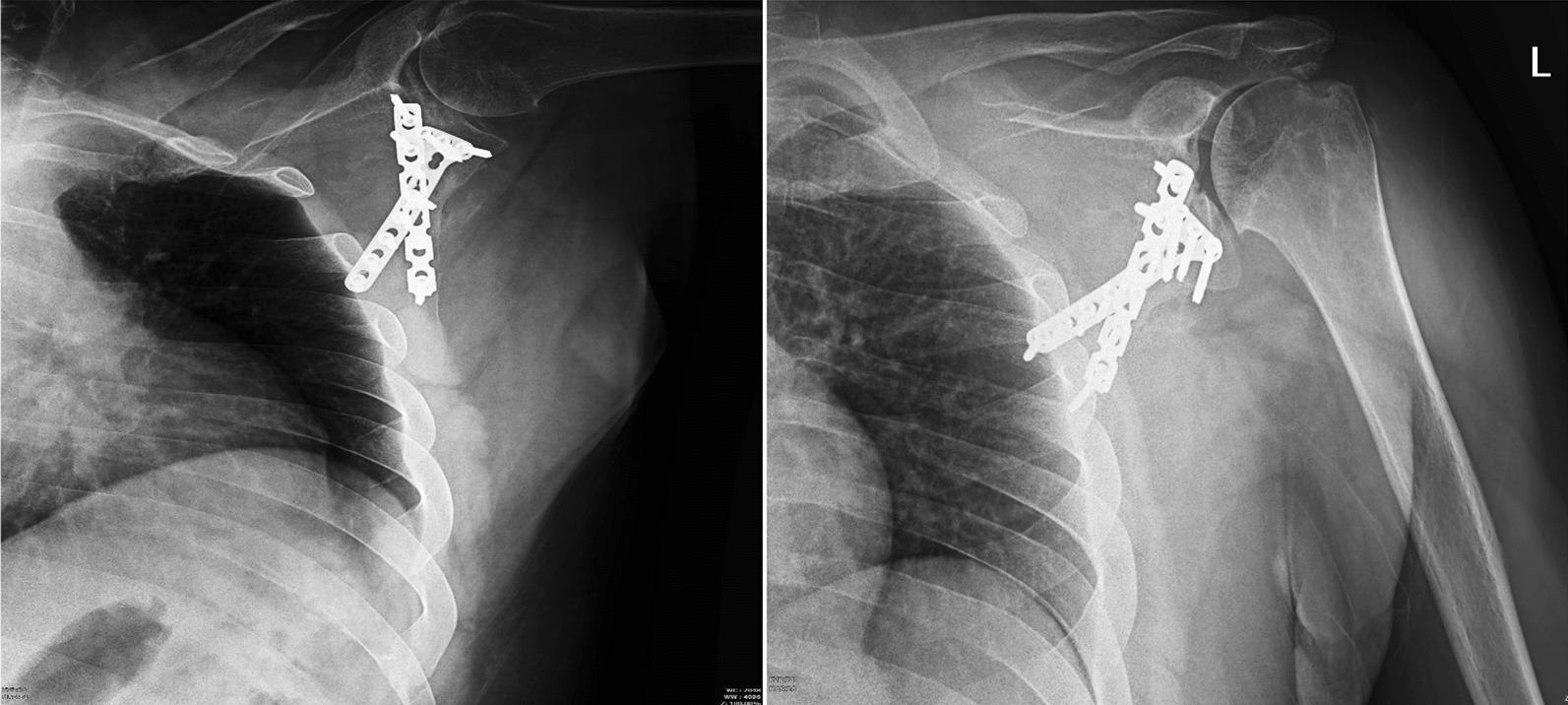
Anteroposterior and lateral views of the shoulder at the 1 year follow-up revealed the complete union of the fracture

Passive movements were initiated on the day following discharge owing to the presence of stable fixation. After 1 month, active movements were performed under the supervision of a physiotherapist for a total of 20 sessions. At the 4 month postoperative evaluation, a radiographic examination was requested for the patient, revealing evidence of complete union. Twelve months after surgery, the patient’s constant score [13] was 79, and shoulder range of motion for abduction, external rotation, and internal rotation, were 100°, 30°, and waist (L3), respectively. The patient reported experiencing pain in stretching the shoulder to its maximum range of motion. His muscle strength was slightly lower than the unaffected side, but he could still abduct his shoulder with up to 15 kg of weight. Figure 7 shows the patient’s 1 year follow-up graph, depicting complete union.

## Discussion

The management of scapula ANFs presents unique challenges, particularly in cases involving morbidly obese patients. In this case report, we outlined our proposed surgical approach, yielding acceptable postoperative outcomes, for a 34-year-old male patient with morbid obesity who experienced an anatomical neck fracture of the scapula following a motor vehicle accident. The delay in surgical intervention due to concurrent life-threatening injuries emphasizes the importance of a multidisciplinary approach and careful consideration of the patient’s overall condition. The utilization of the posterolateral limited Dupont–Evrard approach allowed for adequate exposure and stable fixation of the fracture, taking into account the patient’s high body mass index. Through this case, we contribute to the existing body of literature on scapula neck fractures, highlighting the rarity of radiologically confirmed scapula anatomical neck fractures and the limited evidence regarding their optimal management.

Scapular neck fractures have been the subject of classification systems developed over the years to better understand their anatomical characteristics and guide treatment decisions. In 1913, two types of classifications were presented for scapular neck fractures. Tanton *et al*. categorized scapular neck fractures into three subtypes: anatomical, surgical, and trans-spinous. Similarly, Ada and Miller introduced a classification system in which scapular neck fractures were divided into three types: IIA (surgical neck), IIB (trans-spinous neck), and IIC (infraspinous neck fracture) [14, 15]. These early classifications provided a basic framework for distinguishing between different types of scapular neck fractures.

In 1984, Gagey *et al*. introduced an anatomical classification system for scapular neck fractures, further refining the understanding of these fractures. This classification divided scapular neck fractures into three categories: anatomical neck fracture, surgical neck fracture, and trans-spinous fracture [16]. Building upon the work of Gagey *et al*. and Euler *et al*. another classification system for scapular neck fractures was proposed in 1992. This system classified scapular neck fractures into three categories: C1 (anatomical neck fractures), C2 (surgical neck fractures), and C3 [surgical neck fractures with disrupted superior shoulder suspensory complex (SSSC)] [17]. This classification system took into account the involvement of the SSSC, adding another dimension to the understanding of scapular neck fractures. In 1994 Goss *et al*. introduced a classification system that also included anatomical neck fractures, surgical neck fractures, and infraspinous neck fractures [2]. This classification system distinguished between scapula neck fractures occurring above or below the level of the spine of the scapula. Since the connection between the glenoid fossa and scapular body in the infraspinatus type was intact, we believe this type is a scapular body fracture.

According to the previous studies, the definition of anatomical neck fractures of the scapula in our case aligns with the definition provided by Bartoníček *et al*. [5]. ANFs are characterized by an intact glenoid fossa, with the fracture line extending superiorly to the lateral base of the coracoid while keeping a short inferior spike of the scapular lateral pillar, in less than approximately 4 cm distal to the inferior glenoid pole.

Scapula ANFs are of particular importance due to their tendency toward instability and distinct radiological features that differentiate them from other scapular fractures. Due to the coracoid separation, the only tendon attached to the anatomical neck of scapula is the long head of the triceps brachii, which causes instability of the fragment and increases the GPA [3, 7]. Unlike scapular surgical neck fractures, where the integrity of the SSSC plays a crucial role in the fracture stability, owing to the coracoid’s attachment to the broken piece, in coracoclavicular ligament rupture cases, the fracture becomes even more unstable resulting in reduced GPA, due to the traction exerted by the conjoined tendon, which acts as a strong deforming force [7, 18].

Another challenge associated with scapula ANFs is the small size of the fractured fragment which poses difficulties in achieving stable fixation [2]. This may explain the utilization of various implants reported in previous studies, as surgeons try to find the most appropriate fixation method to address the unique characteristics of these fractures.

To date, the literature on radiologically verified scapular neck fractures remains scarce, with only seven reported cases (Table 1). In 1984 Hardegger *et al*. conducted a study involving 37 patients with scapular fractures requiring fixation, two of which were diagnosed with ANFs [7]. The first case was a 44-year-old male patient whose fracture was managed through the placement of two lag screws. One screw was inserted obliquely from the spine of the scapula to the infraglenoid tubercle, while the second screw protected the inferior tip of the fragment. The second patient, a 24-year-old male, underwent surgery with three screws and one plate. In both cases, the surgical approach employed was the posterior approach through the intermuscular plane of the infraspinatus and teres minor muscles. Following surgery, the postoperative protocol involved immobilization of the patient’s shoulder for 3–5 days, followed by functional rehabilitation. After the follow-up period, both patients were able to resume their ordinary activities without any impairment.

**Table 1:** Characteristics of all eight reported cases of scapula anatomical neck fracture

Case no.	Age	Glenopolar angle	Surgical approach	Utilized implants
1	44	Increased	Judet	Two lag screws
2	24	Increased	Judet	Three lag screws + one buttress plate
3	33	NR	Judet	3.5 mm T-plate
4	NR	Increased	Judet	Two 3.5 mm plates
5	49	Increased	Judet	3.5 mm L-plate + wire loops
6	51	Increased	Judet	T + straight 3.5 mm plates
7	51	Unchanged	Posterior deltoid splitting	Two wires
8 (Our case)	34	Increased (73.9°)	Posterolateral limited Dupont-Evrard	3.5 mm T + reconstruction plates

*NR* not reported

In 1999, Arts *et al*. reported a case of anatomical scapular neck fracture in a 33-year-old man with significant anterior angulation. They fixed the fracture with a 3.5 mm T-plate to the spine of the scapula. Postoperative functional rehabilitation after the operation started with limitation in external rotation and, 3 months after the injury, the patient returned to work without experiencing any symptoms. The authors stated that the concept of floating shoulder as an indication for surgery in scapular neck fractures is limited to specific surgical types, and anatomical neck fractures are inherently unstable, even in the presence of intact clavicle and coracoclavicular ligaments [8].

The fourth scapular anatomical neck fracture was reported by Jeong GK *et al*. in 2005, which was successfully managed by two 3.5 mm plates via the posterior Judet approach [10]. Subsequently, Bartoníček *et al*. reported two cases of this rare fracture in 2013, both resulting from falling from height [3]. The first case was a 49-year-old man, operated with one 3.5 mm plate and wire loops via the Judet approach. Passive movements began immediately after the surgery, followed by the commencement of active movements 6 weeks later. The patient achieved a complete recovery. The second patient was a 51-year-old female with a simultaneous scapular anatomical neck fracture, long head of biceps, and superior labrum avulsion. The fracture was fixed with two plates, one aligned along the lateral border and the other along the spine of the scapula. After 4 weeks of immobilization, rehabilitation was started and the outcome was considered fair with a constant score of 50.

The seventh reported case of scapular anatomical neck fracture was presented by Ogawa *et al*. in 2018 [9], in which a 51-year-old man with a scapular anatomical neck, spine of the scapula, and coracoid process fractures along with acromioclavicular joint subluxation, was operated on with a 7 week delay. The fracture of the scapular spine was stabilized using a reconstruction plate, while the anatomical neck fracture was fixed with two wires. The coracoid fracture remained intact, as it did not exhibit any displacement. Passive movements were started 4 days after the operation, followed by active movements and physical therapy at 5 weeks and 2 months later, respectively. At the 6 month follow-up, the patient demonstrated favorable shoulder movements, with only local tenderness around the plate and mild muscle weakness evident.

To date, various studies reported scapular anatomical neck fractures, but none of them presented radiological evidence of those fractures [9]. According to the anatomical complexity of the scapula, the classification of these fractures should be done based on the findings of the computed tomography scan with three-dimensional reconstruction [5, 19]. A vertical upper fracture line lateral to the coracoid process and a small lower spike are the typical radiological characteristics of ANF. The glenoid fossa is completely separated from the scapula body and coracoid process. Thus, the only tendon attached to the glenoid is the long head of the triceps that is attached to the infraglenoid tubercle. The importance of this issue lies in explaining the instability of these fractures and their radiological features [8]. In our opinion, fractures extending more than 5 cm distal to the infraglenoid tubercle are classified as scapular body fractures, and fractures extending medially to the base of the coracoid are classified as surgical neck fractures [5]. In these types of fractures, additional attachments contribute to the stability of the fracture, such as the teres minor muscle in fractures with long spikes, or coracoclavicular ligament in surgical neck fractures. True scapular anatomic neck fractures typically share the same distal displacement, valgus misalignment, and increased GPA [5].

Due to the unstable nature of ANFs [8], surgery is almost always required to achieve an acceptable reduction, but nonsurgical treatment is an option as suggested by Jan Bartonícˇek for fractures with displacement of less than 1 cm and GPAs between 26° and 55° [5]. Another significant difference between anatomical neck fractures of the scapula and other types is the smaller size of the broken piece, which makes its stabilization more challenging. Various methods such as plate and wire loops or a combination of them have been used to fix these fractures. Based on our review of the literature and the challenges associated with scapular anatomical neck fractures, we propose the use of a stable fixation technique with a plate that allows for early postoperative movements. However, the small size of the glenoid can pose difficulties in achieving optimal fixation. To overcome this challenge, we recommend using scapula lateral border anatomical plates whenever possible. These plates are specifically designed to provide secure fixation in scapular fractures, including anatomical neck fractures. In cases where scapula lateral body plates are not available, alternative options such as a distal humeral Y-type plate or 3.5 mm T-, L-, or precountered reconstruction plates are recommended to place at least three screws into the glenoid fragment and ensure stable fixation.

Another significant challenge we encountered in the treatment of this patient was the delay in fracture surgery. Since scapular fractures, which are displaced to such a degree that surgery is unavoidable, are often caused by high-energy trauma events, it is highly frequent to have injuries to important organs, notably the chest. In such cases, the priority becomes addressing and stabilizing life-threatening conditions, thereby necessitating a delay in the surgical treatment of scapular fractures until the overall condition of the patient improves. On the other hand, due to the fact that the surgical indications of scapula fractures are relatively limited and it is not a familiar procedure for many surgeons, generally these fractures are referred to another facility for treatment, which itself leads to further delays in initiating appropriate management. It is important to note that treatment delays of more than 3 weeks after the initial trauma are considered delayed because scapula fractures exhibit a rapid healing process owing to the well-vascularized muscular envelope surrounding the scapula [20]. Therefore, delayed scapular fracture fixation presents a demanding operation due to the accelerated union rate and soft tissue contraction that occurs during the healing process. In rare situations, even after calluses removal and osteoclasis, achieving anatomical reduction is extremely unlikely due to muscle spasm affecting the fracture fragments. Based on our experience from this case, we highlight the importance of meticulous reduction verification using fluoroscopy to ensure appropriate correction of the glenopolar angle and medialization. This is particularly crucial in the context of delayed surgical intervention for scapular fractures, as achieving anatomical reduction poses challenges, primarily due to the presence of blunt fracture edges.

## Conclusions

This case report sheds light on the challenges associated with managing scapular ANFs in patients with morbid obesity. The delayed surgical intervention, coupled with the utilization of a surgical approach tailored to the patient’s specific anatomical and physiological considerations, proved to be effective in achieving favorable outcomes. The rarity and distinct characteristics of scapula ANFs require a multidisciplinary approach, careful surgical planning, and individualized treatment strategies. The identification of radiological indicators, such as the upper fracture line lateral to the coracoid process, a small lower spike, and an increased GPA, aids in accurately diagnosing these fractures. Surgical intervention is typically necessary due to the inherent instability of scapula anatomical neck fractures. Future studies and long-term follow-up of similar cases are warranted to validate the outcomes presented in this report and to guide clinical decision-making regarding the management of scapula anatomical neck fractures in obese patients.

## Data Availability

The material presented in this study are available from the corresponding author on a reasonable request.

## Abbreviations

ANF: Anatomical neck fracture
BMI: Body mass index
GPA: Glenopolar angle
SSSC: Superior shoulder suspensory complex
